# Rubric for the evaluation of competencies in traumatology in the Degree of Physiotherapy: Delphi approach

**DOI:** 10.1186/s12909-021-02904-4

**Published:** 2021-09-06

**Authors:** Esther Díaz-Mohedo, Rita Romero-Galisteo, Carmen Suárez-Serrano, Esther Medrano-Sánchez, Rocío Martín-Valero

**Affiliations:** 1 Faculty of Health Sciences, Department of Physiotherapy, University of Málaga, C/ Arquitecto Francisco Peñalosa, 3, 29071 Málaga, Spain; 2 Faculty of Nursey, Physiotherapy and Podiatry, Department of Physical Therapy, University of Sevilla, C/ Avicena, s/n, 41009 Seville, Spain

**Keywords:** Evaluation, Higher education, Physiotherapy, Rubric, Delphi

## Abstract

**Background:**

In health professions, the curriculum that must be met in order to obtain the academic certificate is based on the development of the so-called *competencies*. The broad content of the Practicum of the Degree of Physiotherapy has led to the creation of multiple types of evaluation, which makes it difficult for faculty members to reach a consensus on competencies. The aim of this study was to develop and validate content of a rubric for the evaluation of acquired competencies related to physiotherapeutic performance and intervention in traumatology within the Practicum of the Degree of Physiotherapy.

**Methods:**

Following the Delphi methodology, a group of experts from all over the Spanish territory participated in the study. Through on-line questionnaires, several sequential rounds were established, alternated by controlled feedback until obtaining a consensus in the opinion of the experts, which allowed elaborating the final rubric.

**Results:**

Initially, 16 experts were contacted, of whom 10 worked and completed the final content of the rubric. For the 3 rounds that were conducted, the initial 142 interventions of the initial proposition, which correspond to specific competencies, were reduced to the final 29 items that compose the specific evaluation rubric presented in this study.

**Conclusions:**

This rubric is an evaluation instrument with valid content for the assessment of specific competencies of Traumatology in the Practicum of the Degree of Physiotherapy.

## Background

In health professions, the curriculum that must be met in order to obtain the academic certificate is based on the development of the so-called *competencies*. These are defined not only as the understanding of the content, problem solving, clinical abilities and attitudes; they also include the *know-how* (or procedural knowledge) in the professional context, which requires the student to implement the acquired knowledge in a creative, flexible and responsible manner [1]. In Europe, higher education is usually standardised though the Bologna Process [2, 3]. This challenge of homogenising the teaching-learning procedures involves the use of a common language, which has resulted in the proposition of contents and tools from different disciplines to respond to such a goal [4, 5].

At the same time, the evaluation of the abilities acquired by the undergraduate students of the Degree of Physiotherapy during their university education has generated multiple publications in the last years, which have provided different tools designed to assess the competencies acquired in different areas, such as patient education [5], clinical performance [6] and interprofessional collaboration [7].

Despite the existence of other, widely studied evaluation tools [8, 9], the convenience of having consensual guidelines to score and evaluate the learning of the students has popularised the use of rubrics. These are useful to examiners, instructors and students [10]. In Physiotherapy, the evaluation of the clinical practices within the Practicum subject is varied, since their content in the different fields is heterogeneous, which generates a lack of consensus in the scientific literature about the contents that must be evaluated in the different areas [11]. In addition to this, a large number of the evaluation tools that are currently used do not comply with the adequate psychometric properties [12, 13]. In the evaluation of the Practicum of the Degree of Physiotherapy (an eminently practical subject, developed in the different health centres), such lack of consensus is evident in the specific assessment of the competencies in Traumatology [14].

Consensus methods, such as Delphi, allow synthesising information about a specific problem. Sequential rounds, alternated by controlled feedback, are used with the aim of reaching a consensus in the opinion of a group of identified experts [15]. Therefore, this is a useful approach in situations where individual judgements must be considered to address an incomplete state of knowledge.

The aim of this study was to develop and validate the content of a rubric for the evaluation of acquired competencies related to physiotherapeutic performance in Traumatology within the Practicum of the Degree of Physiotherapy.

## Methods

The Delphi methodology was followed, which is useful and widely employed to identify and clarify roles, to reach consensus and to synthesise information, both in medical field and education, allowing the participation of an identified group of experts within the Spanish territory [5].

### Participants

Using a purposive sampling approach [16], 16 potential-participant experts were identified by members of the research team and were subsequently contacted and invited to participate in the Delphi questionnaire via e-mail. For these clinical consensus studies, Jones and Hunter recommend the participation of specialists in the specific area [17].

In this study, an *expert* was defined as a physiotherapist with over 5 years of experience as either a faculty member in the Practicum subject of the Degree of Physiotherapy, as a clinical tutor participating in the Practicum, or as a healthcare professional specialised in the field of Physiotherapy and specifically in Traumatology. The *expert* was also required to be working within the Spanish territory.

 All participants signed informed consent documents. The study was approved by the Ethics Committee of the University of Málaga (CEUMA: 34-2020-H).

### Procedure

This study incorporated three rounds of on-line questionnaires, described in the next section, which proved to the sufficient to generate an adequate feedback and establish a broad consensus on different opinions. New rounds were planned to accommodate additional interventions or other problems that could justify this research. Each round of questionnaires was open for two weeks and a reminder e-mail was sent to all participants who had not replied two days before the submission deadline.

### First round

The questions of the first round were formatted in the on-line software LimeSurvey and were sent through a link via e-mail to each participant. The first Delphi round consisted of two sections.

In the first section, socio-demographic information of the experts was requested, including gender, age, professional area, professional experience in years and geographic location. This information was used to obtain feedback about the structure of the expert panel and guarantee the heterogeneous flow of contributions with regard to the analysis of the data.

In the second section, the experts were asked the open-ended Delphi question, developed by the authors of the study with the specific aim of generating enough themes integrated in the answers of the panel members, in line with a Delphi approach [5].

The initial question was designed to direct the experts toward the consideration of multiple competencies and the consideration of competencies that physiotherapists may or may not have regarding physiotherapy applied in the field of Traumatology: “ What practical interventions do you think that undergraduate Physiotherapy students should carry out during their clinical practice in order to respond to the specific competencies related to the contents of Physiotherapy in Traumatology within the Practicum subject?”.

Based on the answers obtained, a search was conducted in different databases to identify possible professional interventions in this field. The terms used were “physiotherapy”, “traumatology”, “competencies”, “assessment”, “evaluation”, “skills”, “knowledge” and “methods”. This literature review allowed completing the identification of different categories/areas of competencies that had to be addressed to improve the validity of the content of this tool.

The editing of the obtained feedback was slightly modified to make it clearer. The 44 interventions into which the initial 142 interventions were summarised are the following:

### Specific

- Systematically elaborate and complete the physiotherapeutic medical record.
- Correctly record and write down the most relevant aspects of the evaluation of the patient: anamnesis, inspection, observation, examination, extraction and interpretation of data from medical reports and complementary diagnostic tests.
- Know the generalities of the most common medical-surgical interventions in the scope of traumatology, as well as the cicatrization time of the different tissues.
- Identify as many symptoms and signs reported by the patient, as well as the psychosocial risk factors that may influence his/her recovery.
- Identify yellow and red flags or alarm signals that could require referral to a specialist during the patient’s evaluation/treatment process.
- Perform a complete evaluation of the patient’s movement and its possible alterations, making use of the pertinent measurement instruments (goniometer, measuring tape, stabilizer, etc.), recording such results adequately.
- Carry out a complete evaluation of the patient’s gait and its possible alterations.
- Assess and record pain through validated instruments and identify the type of pain presented by the patient (nociceptive, visceral, neuropathic or chronic/dysfunctional).
- Conduct a diagnostic palpation that allows you to obtain information of local suffering points, tissue normality and alterations of the palpated area, tissue asymmetries, etc., with anatomical precision and pressure adapted to the depth of the evaluated Sec. 
- Carry out a neurological assessment of the patient through a test of superficial and deep sensitivity and reflexes, recording such information with specific terminology and interpreting the results adequately.
- Correctly select and perform orthopedic and/or functional tests, and interpret the results adequately.
- Write down the report of the patient’s physiotherapeutic evolution and/or discharge.
- Be capable of clinical reasoning, formulating coherent hypotheses and proposing differential diagnosis strategies that allow you to correctly identify the dysfunction presented by the patient and establish the physiotherapeutic diagnosis according to the internationally accepted rules.
- Correctly propose coherent physiotherapeutic objectives for the short, medium and long term, considering the pathology and the individuality of the user and his/her expectations and preferences.
- Plan the treatment in coherence with the objectives set, attending to the adequacy, validity and efficiency criteria, considering risks and contraindications, and efficiently managing the treatment time.
- Explain to the patient the purpose of the therapeutic interventions ensuring the adherence to the treatment.
- Reevaluate results periodically and adapt the intervention plan.
- Correctly execute a functional (preventive and/or therapeutic) and neuromuscular bandaging, knowing how to choose among the different materials and techniques to achieve the best result based on the objective set.
- Correctly execute a compressive bandaging to treat the edema.
- Correctly execute the procedure to calculate the TI curve in a certain muscular group and correctly interpret the result to subsequently proceed with electrostimulation.
- Know the effects of an electrotherapeutic application (motor electrostimulation, TENS, galvanic current, magnetotherapy, laser, shockwave therapy), know how to apply it correctly, selecting among the different parameters, establishing dosimetries and application times, etc., to achieve the best result based on the objective set.
- Know the effects of the different types of active exercise (isometric, concentric and eccentric isotonic, functional, motor control, etc.), adequately choosing among them and prescribing and controlling their correct execution to achieve the best result according to the objective set in a musculoskeletal dysfunction.
- Know the principles of proprioceptive reeducation in its different phases, adequately selecting the techniques depending on the evolution phase of the patient, and perform, prescribe and control its execution to achieve the best result according to the objective set.
- Know the effects of an application of thermotherapy (paraffin baths, microwaves, short wave, ultrasound, tecar therapy, etc.), know how to apply it correctly, selecting among the different parameters, establishing dosimetries and application times, etc., to achieve the best result according to the objective set.
- Know the effects of support and loading in lower limb injuries, and know how to carry out the procedure of progressive loading and gait reeducation for the patient.

-  Know the effects of the following manual therapy techniques, adequately selecting among them and executing them correctly to achieve the best result according to the objective set:

26. - Masotherapy,
27. - Neuromuscular techniques,
28. - Myofascial techniques,
29. - Articulatory techniques,
30. - Manipulative techniques,
31. - Neurodynamic techniques,
32. - Dry needling techniques,
33. - Mulligan techniques,
34. - Sohier techniques.
35. - Know and apply cortical reorganisation techniques (motor imagery, mirror therapy, gradual exposure to movement).
36. - Know the indications and contraindications of all the above described therapeutic interventions.
37. - Know, design and implement Patient Health Education programmes in different situations (chronic diseases, pain, risk groups, etc.)
38. - Take into account safety measures, such as hand washing, use of gloves and protecting injured areas (depending on the patient’s evolution phase).
39. - Know and use relaxation techniques.

### Transversal

40. - Show verbal and non-verbal communication skills, as well as an active listening attitude.
41. - Show empathy for the patient and face conflict situations adequately.
42. - Use an appropriate vocabulary in all contexts: patient, family and interdisciplinary team.
43. - Be active and proactive in the workplace, and interact correctly with the rest of the team members.
44. - Be responsible (punctual, correctly identified and uniformed, comply with the internal functioning rules of the centre, the data protection law, rotation dates and timetable/schedule, take care of the material, etc.), reflect on the risks and consequences of your interventions, and communicate with the manager in the face of any event.

### Second round

The open questions of the first round were subjected to a frame analysis, as recommended by the Delphi approach [18]. The principal investigator, who was qualified for the use of qualitative research methods, read all the data several times to familiarise with the meanings that were attributed to the practical competencies and the corresponding practical interventions. Each potential theme was discussed by the research team. An initial list of 142 interventions was synthesised and reduced to 39 items by the research team, based on a tentative list from different sources: competencies defined by the Ministry of Education for the Degree of Physiotherapy, statements about competencies from the White Paper about the Physiotherapy degree in Spain [19]. In compliance with Order CIN/2135/2008, the requirements for the verification of official university titles that enable a person to work as a physiotherapist were established and grouped into three dimensions: clinical history in Physiotherapy, physiotherapeutic diagnoses and clinical reasoning in traumatology. The final 39 items were written in the present tense, according to the design of the intervention, and they were presented in a format that allowed being verified by the expert panel [5]. Each member of the panel of the first round received an e-mail directly from the principal investigator with a link to the second questionnaire with the 29 items that corresponded to the different competencies. The experts were asked to express, independently, their degree of conformity with the statements of each of the item proposed by all of them. The answers were established in a 5-point Likert scale, from 1 (totally disagree) to 5 (totally agree). A final space was included, in which the experts could leave a comment that could help to identify additional competencies-interventions which they believed were not included or to point out any problems they detected among the 39 items provided.

The analysis of the Delphi panels was performed using descriptive statistics that included measures of central tendency (median) and dispersion (interquartile range). Consensus was defined as the 75th percentile or higher values in the score of each item obtained by the panelists and an interquartile range below 3.

The interventions that did not reach a consensus or needed to be reformulated with respect to the items in this round were the following:

- Understanding the generalities of the medical-surgical interventions that are most commonly attended to in the field of Traumatology, as well as the wound healing times of the different tissues.
- Identifying most of the symptoms and signs reported by the patient, as well as the psychosocial risk factors that may influence his/her recovery.
- Conducting a complete evaluation of the gait of the patient and its possible alterations.
- Conducting a diagnostic palpation that allows obtaining information of local pain points, tissue normality and alterations of the examined area, tissue asymmetries, different sensations, etc., with anatomic precision and pressure adapted to the depth of the assessed plane.
- Knowing the effects of the following techniques of Manual Therapy, selecting adequately among them and executing the correctly in order to obtain the best result based on the objective set: Myofascial Techniques, Manipulation Techniques, Dry Needling Techniques, Mulligan Techniques and Sohier Techniques.
- Knowing and applying different cortical reorganization techniques (motor imagery, mirror therapy and graded exposure to movement).

### Third round

Each member of the panel received a new e-mail with a link to the second questionnaire with the resulting 30 items that corresponded to the different competencies. They were asked to proceed in the same manner, i.e., expressing their degree of conformity with the statements of the items-interventions on which they all agreed, using the same 5-point Likert scale, again with an additional open space to leave any comments and/or suggestions.

The answers of the third round were analysed based on the same stabilization criteria, resulting in a final rubric with 29 items.

## Results

### Expert panel

 Of the 16 experts that were invited to participate, 10 accepted the invitation and signed an informed consent form. These 10 responded to all three rounds (10/10, 100 %). The demographic characteristics of the expert panel are presented in Table 1.

**Table 1:** Demographic characteristics of the expert panel

	Gendern (%)	Workplace n (%)	Years of teaching experiencen (%)	Years of clinical experiencen (%)	Age (years)n (%)
Female	3 (30 %)				
Male	7 (70 %)				
U. of Castilla la Mancha		2 (20 %)			
U. of Jaén		1 (10 %)			
U. of Sevilla		1 (10 %)			
U. of Valencia		2 (20 %)			
U. of Ponferrada		1 (10 %)			
U. of Granada		1 (10 %)			
U. of La Coruña		1 (10 %)			
U. of Málaga		1 (10 %)			
0 to 10			3 (30 %)		
11 to 20			4 (40 %)		
21 to 30			2 (20 %)		
31 to 40			1 (10 %)		
0 to 10				4 (40 %)	
11 to 20				4 (40 %)	
21 to 30				1 (10 %)	
31 to 40				0 (0 %)	
41 to 50				1 (10 %)	
20–30					1 (10 %)
31–40					2 (20 %)
41–50					4 (40 %)
51–60					1 (10 %)
61–70					2 (20 %)

*U.* University

#### Summary of the Delphi process

**Fig. 1:**
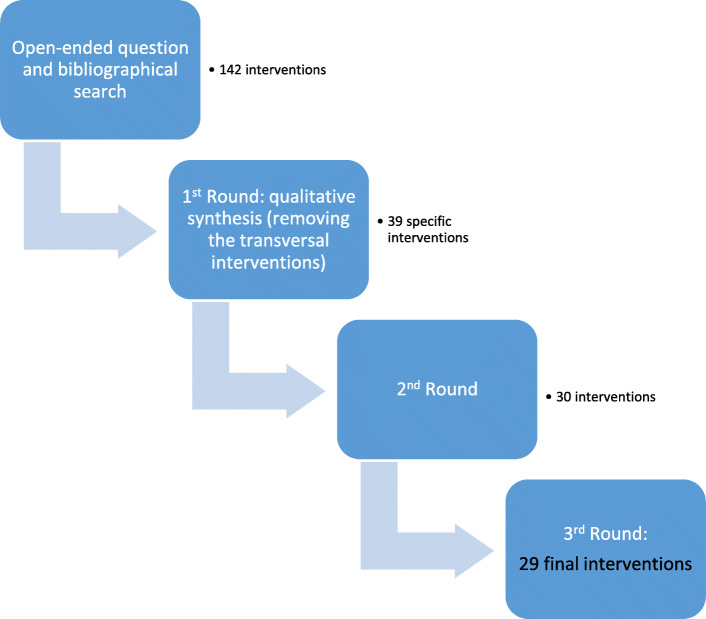
Summary of the procedure by rounds

The Delphi phase required 3 rounds to reach consensus (Fig. 1). This method produced a list of 39 interventions that were associated with each of the 13 competencies (Table 2).

Of the 39 interventions provided in the second round, 30 were agreed on and presented in the third round, in which the experts agreed upon 29 interventions that they finally considered important to include in the rubric of the Practicum of Physiotherapy to respond to the specific competencies related to Physiotherapy in Traumatology (Fig. 1).

The final interventions and their corresponding specific competencies are shown in Table 2.

**Table 2:** Competencies and their association with the interventions

Specific competencies of the White Paper about the Degree of Physiotherapy	Interventions of the rubric
1. Elaborate and complete the clinical physiotherapy history.	Elaborate and complete the clinical physiotherapy history in a systematic manner
	Correctly record and write the most relevant aspects of the valuation of the patient: anamnesis, inspection, observation, exploration, extraction of data of medical reports and diagnostic tests
2. Examine and assess the functional state of the patient/user.	Identify alarm signs that may influence the evolution of the treatment and/or may require referral to the specialist during the evaluation/treatment of the patient
	Perform, in patients who require so, an evaluation (visual and/or instrumental) of the movement of the patient (range, quality, strength,…) and its possible alterations, recording such results adequately
	Assess and record pain, in patients who require so, through validated instruments, and identify the type of pain observed (nociceptive, visceral, neuropathic or chronic-dysfunctional)
	When appropriate, conduct a neurological assessment of the patient through a superficial and deep sensitivity test and a reflex test, recording such information with proper terminology and interpreting the results adequately
3. Determine the physiotherapy diagnosis.	Identify most of the symptoms and signs reported by the patient
	Correctly select and conduct orthopedic and/or functional tests and adequately interpret the results
	Be able to perform clinical reasoning, proposing coherent hypotheses and strategies of differential diagnosis
4. Design the physiotherapy intervention or treatment plan.	Correctly prescribe and control physical exercise in its different modalities (isometric, concentric and eccentric isotonic, functional, motor control, conscious movement, etc.), adequately selecting among them to achieve the best result according to the objective set in a musculoskeletal dysfunction
	Prescribe, perform and/or control the Proprioceptive and Neuromuscular Re-education Techniques in their different phases, adequately selecting the techniques according to the evolutionary phase of the patient to achieve the best result based on the objective set
5. Execute, direct and coordinate the physiotherapy intervention plan.	Correctly propose coherent physiotherapeutic objectives in the short, medium and long term, considering the pathology and the individuality of the user and his/her expectations and preferences
	Plan the treatment based on the objectives set, attending to the criteria of adequacy, validity and efficiency, considering risks and contraindications and efficiently managing the treatment time
	Correctly perform a bandaging (functional, neuromuscular, compressive), knowing the best choice among the different materials and techniques to achieve the best result according to the objective set
	Correctly carry out an application of electrotherapy (motor electrostimulation, TENS, galvanic current, magnetotherapy, laser, shock waves), selecting among the different parameters, and establishing dosimetries and application times, etc., to achieve the best result according to the objective set
	Know the effects of the different techniques of Manual Therapy: massage therapy (decontraction, bowel evacuation, cicatrization massage, Cyriax), adequately selecting among them and correctly executing them to achieve the best result according to the objective set
	Correctly carry out an application of thermotherapy (paraffin baths, MW, PSWT, radiofrequency, etc.), selecting among the different parameters, and establishing dosages and application times, etc., to achieve the best result according to the objective set
	Carry out the procedure of progressive loading and gait reeducation in patients who require so due to the lower limbs injuries
	Correctly carry out the following techniques of Manual Therapy: neuromuscular techniques, adequately selecting among them to achieve the best result according to the objective set
	Correctly carry out the following techniques of Manual Therapy: articulatory techniques, adequately selecting among them to achieve the best result according to the objective set
	Correctly carry out the following techniques of Manual Therapy: neurodynamic techniques, adequately selecting among them to achieve the best result according to the objective set
6. Motivate others.	Explain to the patient the purpose of the therapeutic interventions to achieve their adherence to the treatment
7. Evaluate the evolution of the results.	Re-evaluate the results periodically and adapt the intervention plan
8. Provide an efficient and integral care.	In patients who require so, apply techniques of cortical reorganization (motor imagery, mirror therapy, graded exposure to movement)
9. Incorporate scientific research and evidence-based practice as professional culture.	Know the indications and contraindications of all the proposed therapeutic interventions
10. Intervene in health promotion and disease prevention.	Know, design and implement educational programmes for the health of the patient in different situations (chronic diseases, pain, risk groups, etc.)
11. Write the physiotherapy discharge report.	Write the physiotherapy report of the progress and/or discharge of the patient
12. Keep knowledge, abilities and attitudes up to date.	Consider safety measures such as handwashing, the use of gloves and protection of the injured areas (based on the progress period)
13. Manage stress.	Know and use relaxation techniques in patients who require so

The intervention that did not reach the consensus of the expert panel was the following:

- Correctly executing the procedure to calculate the time-intensity curve (TIC) in a specific muscular group and correctly interpreting the result for the subsequent electrostimulation procedure.

## Discussion

In this study, we developed and validated the content of a rubric for the evaluation of specific competencies related to physiotherapeutic performance and intervention in the field of traumatology for use by undergraduate Physiotherapy students in the corresponding Practicum. After the analysis of the initial 142 interventions, a final evaluation rubric with 29 interventions, corresponding to 13 specific competencies, was agreed upon by 10 experts.

The current certification state of university titles generates the need to create competency-based evaluation tools that guarantee the quality of the evaluation processes based on the direct observation of the different interventions of the students with real patients in the clinical context. The evaluable criteria must consider those tasks from the cognitive, procedural, affective and interpersonal perspectives, including the evaluation of the different interventions conducted. These range from the integral approach applied in the first contact with the patients/users of the health system to the diagnoses and the different therapeutic and preventive interventions carried out [20].

The evaluation performed during the university practical training helps students to develop abilities that will allow them to establish an adequate relationship with their patients [21]. Previous studies claim that rubric-based evaluations are equivalent to and possibly more accurate than the traditional evaluation methods (clinical evaluation of health professionals) in the improvement of the knowledge and abilities acquired through education by the students of health professionals [22].

There is a large variety of instruments designed to evaluate university students during their training period [5, 8]. However, the results of a systematic review criticise the lack of evidence about their educational impact and the evaluation of the educational results [6].

Some studies support the qualitative approach, which, through exhaustive interviews and focus groups, provide an enriching perspective for the identification of the thematic categories about the competencies of a specific field within physiotherapy to select the clinical interventions of the experts [23]. Other authors use quantitative methods, such as questionnaires [24], application of the Generalizability Theory [25] and mixed approaches [7]. The Delphi method for information gathering used in the present study is the most widely used in the field of Health Sciences [26, 27]. However, the issues identified in this study are dynamic and may change over time as the practice of mindfulness progresses and may need to be revisited in the near future [28].

The rubrics for the evaluation of competencies acquired by the students in health university subjects are of objective utility to determine their critical thinking, case-resolution capacity, etc. through the positive feedback obtained [29], which helps constituting such rubrics as a potentially valuable strategy that must be implemented from higher education. The implementation of these tools must be based on a multidimensional theoretical framework such as the one used in this study, thus providing a high content validity. The set of selected interventions was constantly subjected to the competencies that they had to represent, that is, the traumatology competencies that should be included in order to: elaborate and complete the clinical physiotherapy history; examine and value the functional state of the patient/user; determine the physiotherapy diagnosis; execute, direct, design and coordinate the physiotherapy intervention plan; evaluate the evolution of the results, etc. [19].

Our results are in line with those of previous studies, which claim that the use of a rubric facilitates the evaluation of the clinical reasoning of Physiotherapy in Traumatology [14, 25]. We believe that the implementation of the rubric-based evaluation tool has increased the accuracy of the evaluation of physiotherapy students with respect to the clinical professionals objective score [30, 31].

Strengths: Traditionally, clinical competencies have been assessed frequently through general impressions from repeated encounters between professionals, clinical tutors and students, with obvious limitations due to heterogeneity and lack of assessment criteria. The findings of this study can help all stakeholders to solve this problem.

Limitations:

Given that the Delphi process was conducted in an asynchronous manner, the experts and the researchers had limited opportunity to resolve any miscommunications. Although it is deemed a valuable method to generate knowledge in an area that lacks empirical evidence, the competencies selected in our study should be subjected to further research to investigate their usefulness across different clinical settings and within assessment measures [5].

Further research should also explore how individual competencies may be grouped or categorised based on the clinical setting or stage of the patient consultation. This should be validated through consensus or observational research to further develop a competency model that is consistent with relevant frameworks for physiotherapists and aid in practical applications.

## Conclusions

This rubric is expected to contribute to improving the content validity of an instrument that will be used to assess the competencies of undergraduate Physiotherapy students in the field of Traumatology in the Practicum. Both students and faculty members will have at their disposal an optimised and recent instrument that removes subjectivity in the evaluation process, clearly defining what is going to be evaluated. Furthermore, this tool will help to internalise in faculty members and students the specific competences of Traumatology in the Practicum of the Degree of Physiotherapy.

This study demonstrates the feasibility of using expert consensus to establish conditions impacting the complexity of procedural skills, and the benefits of incorporating the Delphi method.

## Data Availability

The dataset used during the current study is available from the corresponding author on reasonable request.
